# Ventricular access device placement in the fourth ventricle to treat malignant fourth ventricle brain tumors: technical note

**DOI:** 10.1007/s00381-015-2969-y

**Published:** 2015-11-23

**Authors:** David I. Sandberg, Marcia L. Kerr

**Affiliations:** Division of Pediatric Neurosurgery, Departments of Pediatric Surgery and Neurosurgery, University of Texas Health Science Center at Houston and Mischer Neuroscience Center, 6431 Fannin Street, MSB 5.144, Houston, TX 77030 USA; Divisions of Neurosurgery and Pediatrics, University of Texas MD Anderson Cancer Center, 6431 Fannin Street, MSB 5.144, Houston, TX 77030 USA; Division of Pediatric Neurosurgery, Department of Pediatric Surgery, University of Texas Health Science Center at Houston, 6431 Fannin Street, MSB 5.144, Houston, TX 77030 USA

**Keywords:** Brain tumor, Fourth ventricle, Ventricular access device, Local delivery, Intraventricular, Ommaya

## Abstract

**Purpose:**

Ventricular access devices (VADs) are commonly placed in the lateral ventricle but rarely placed in other ventricular compartments. This manuscript describes technical aspects of VAD placement into the fourth ventricle for the purpose of treating malignant posterior fossa brain tumors.

**Methods:**

As part of a pilot clinical trial to treat recurrent malignant brain tumors in children, seven patients underwent posterior fossa craniotomy and placement of a ventricular catheter under direct vision into the fourth ventricle. The catheter was placed without passing through any brain parenchyma. It was then connected to a VAD placed subcutaneously at the inferior aspect of the incision. Three of the seven patients underwent simultaneous subtotal resection of recurrent tumor located in the fourth ventricle or cerebellum, and one patient underwent simultaneous tumor biopsy. The VAD was used to administer chemotherapy (methotrexate) in five of the seven patients.

**Results:**

Six patients had no new neurological deficits after surgery, and one patient had partial left-sided facial weakness that was attributed to resection of tumor close to the floor of the fourth ventricle. No new neurological deficits were caused by VAD placement or by methotrexate infusions into the fourth ventricle.

**Conclusions:**

A VAD for chemotherapy infusion can be placed safely into the fourth ventricle without damaging the brainstem or cerebellum. Attention to anatomical details specific to the fourth ventricle are important when placing a fourth ventricle VAD and when using it to administer chemotherapy.

## Introduction

Infusion of chemotherapy directly into the fourth ventricle of the brain is a novel means of treating malignant posterior fossa brain tumors that originate in the fourth ventricle. In a recently published pilot clinical trial [1], seven patients with recurrent, malignant posterior fossa tumors underwent implantation of a ventricular access device (VAD) via a posterior fossa craniotomy. Five of these seven patients received repeated methotrexate infusions into the VAD without any recognized neurological toxicity, and all three patients with medulloblastoma had decreased tumor burden after infusions. The promising results of this trial have led to additional clinical trials testing infusion of chemotherapy (clinical trials.gov ID NCT 02458339) and expanded autologous natural killer cells (clinical trials.gov ID NCT 02458339) that both recently opened.

VAD placement into the lateral ventricle of the brain is a common procedure that is familiar to neurosurgeons. Prior to the recent pilot trial at our institution [1], placement of a VAD into the fourth ventricle for infusion of chemotherapy had not been previously reported. The purpose of the current manuscript is to detail, for a neurosurgical audience, the technical nuances required to safely place and use a VAD in the fourth ventricle.

## Methods

Fourth ventricle catheters attached to a VAD were placed in seven patients enrolled in a pilot clinical trial (clinicaltrials.gov ID NCT01737671) for infusion of methotrexate into the fourth ventricle in patients with recurrent, malignant posterior fossa tumors [1]. Prior to initiation of the study, Investigational New Drug (IND) exemption was obtained from the FDA (IND Exemption Number 116804) and institutional review board approval was obtained from the University of Texas MD Anderson Cancer Center (Protocol 2012-0823) and Children’s Memorial Hermann Hospital/University of Texas Health Science Center at Houston (Protocol HSC-MS-12-0492). Informed consent was signed by all patients and/or their legal guardians.

### Surgical technique for placement of fourth ventricle catheter and VAD

**Fig. 1:**
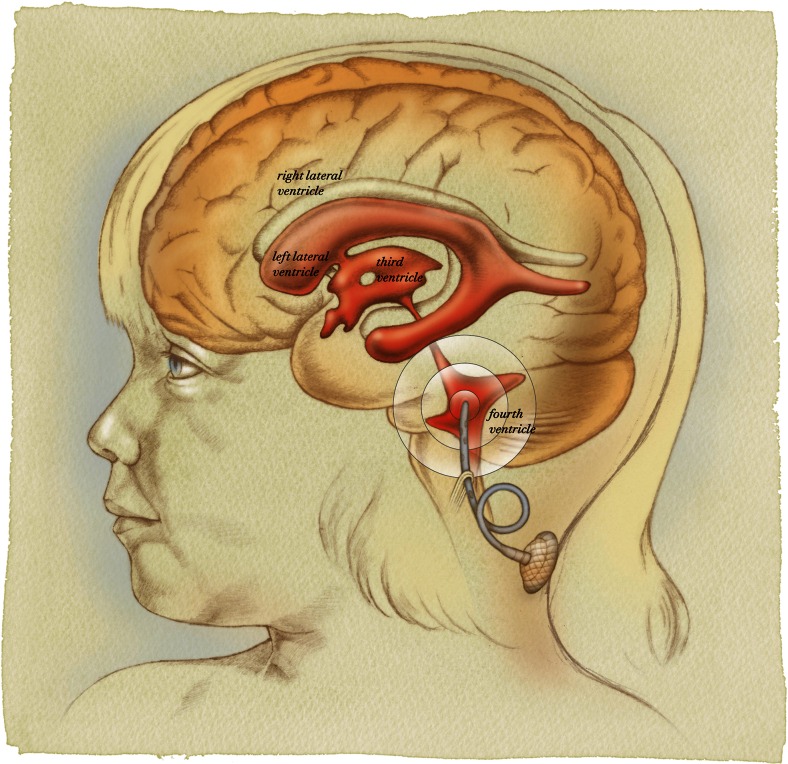
Artist’s illustration demonstrating catheter position in the fourth ventricle parallel to the floor of the fourth ventricle. The catheter is secured to dura or soft tissue inferior to the dural closure and then has a relief loop in place to avoid tension on the catheter with neck movement. It is then attached to a ventricular access device (VAD) that is tunneled subcutaneously at the inferior aspect of the incision

Posterior fossa exposure is obtained via a standard midline suboccipital craniotomy. If resectable tumor is present within the operative field, maximal safe surgical resection is performed under the operating microscope using standard microsurgical techniques. After hemostasis is obtained, a ventricular catheter is placed into the fourth ventricle and attached to a VAD placed subcutaneously at the inferior aspect of the incision as illustrated in Fig. 1.

**Fig. 2:**
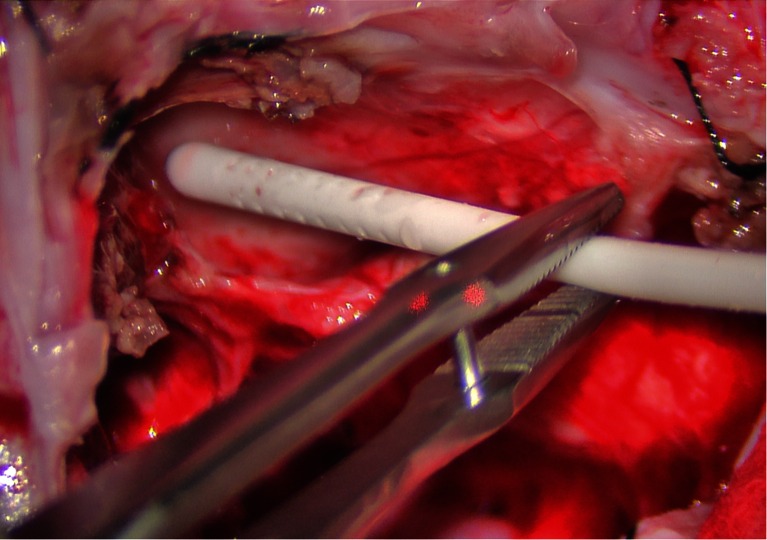
Intraoperative image demonstrating catheter placement into the fourth ventricle. The catheter is placed under the operating microscope under direct vision without passing through any brain parenchyma

**Fig. 3:**
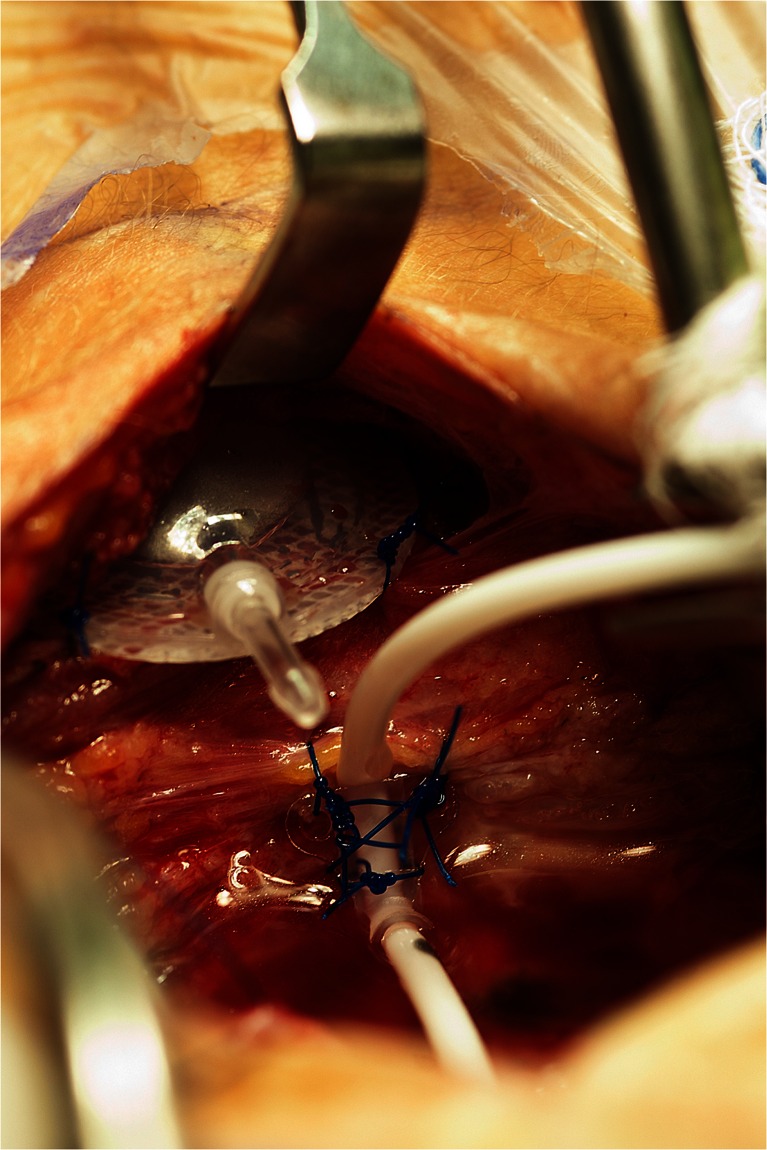
Intraoperative image demonstrating VAD that has been placed subcutaneously at the inferior aspect of the incision and secured to underlying fascia with 3-0 prolene sutures. The VAD will be connected to the catheter that has been placed and secured to soft tissue and/or dura at the inferior aspect of the dural opening

To safely place the ventricular catheter into the fourth ventricle, the catheter is placed under direct vision using the operating microscope without passing through any brain parenchyma (Fig. 2). Great care must be taken to ensure that the catheter is positioned and secured in a manner that maintains its position parallel to the long axis of the fourth ventricle to avoid injury to the brainstem or cerebellum. The catheter is placed such that all catheter holes are within the fourth ventricle, and the catheter tip is well short of the cerebral aqueduct to avoid injury to the periaqueductal gray. Once the catheter is in final position, it is secured with 3-0 prolene sutures in several placed to soft tissue and/or dura at the inferior aspect of the dural opening using the right angle clip provided with the ventricular catheter (Fig. 3).

**Fig. 4:**
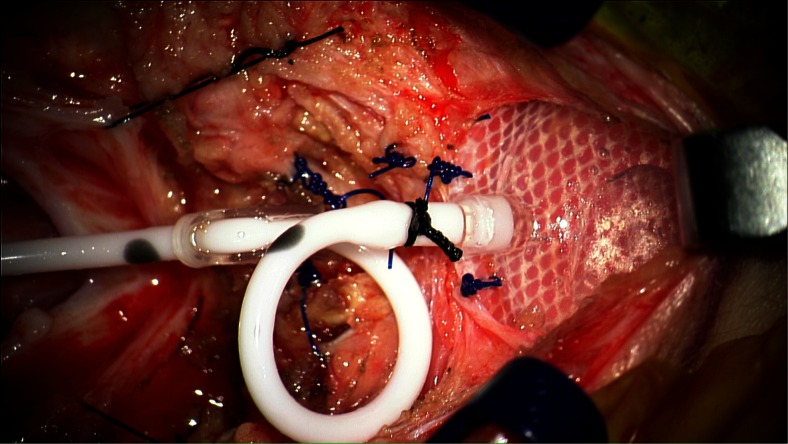
Intraoperative image demonstrating catheter secured to VAD. A relief loop is placed to minimize tension on the catheter with neck movement

After creating a small pocket for the VAD at the inferior aspect of the incision with a hemostat, the VAD is inserted into the pocket and secured to the underlying fascia with several 3-0 prolene sutures (Fig. 3). The catheter is cut to the appropriate length to allow for a relief loop before attachment to the VAD (Fig. 4). The relief loop serves to minimize tension on the catheter with subsequent neck movement postoperatively. When sizing the catheter appropriately for the relief loop, it is important to make sure that catheter will be maintained in this position without any kinking. A 2-0 silk suture is used to secure the catheter to the VAD.

**Fig. 5:**
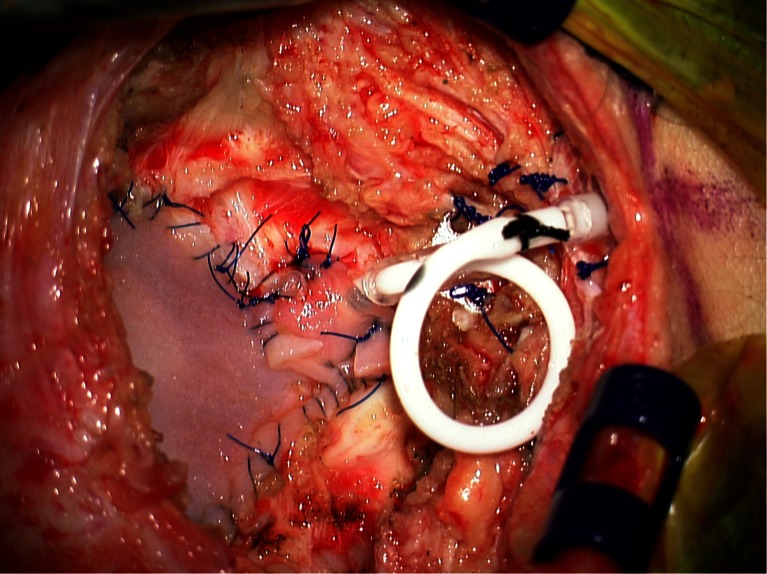
Intraoperative image demonstrating dural closure using duraplasty with allograft. After initial duraplasty, an additional small piece of allograft is sutured at the inferior aspect of the dural closure to minimize the possibility of cerebrospinal fluid egress while ensuring that the catheter is not occluded

After the catheter has been secured to the VAD, a duraplasty is performed either with pericranium, if still present after previous surgeries, or with an allograft (Fig. 5). When performing the duraplasty, great care must be taken when suturing the dural graft around the catheter at the inferior aspect of the dural closure. The dural closure must be tight enough to minimize the possibility of cerebrospinal fluid (CSF) egress but cannot be so tight that it occludes the catheter. If necessary, as illustrated in Fig. 5, additional pieces of dural graft, pericranium, or fascia are sutured to the inferior aspect of the dura to accomplish this goal.

### Postoperative imaging prior to using the fourth ventricle VAD

**Fig. 6:**
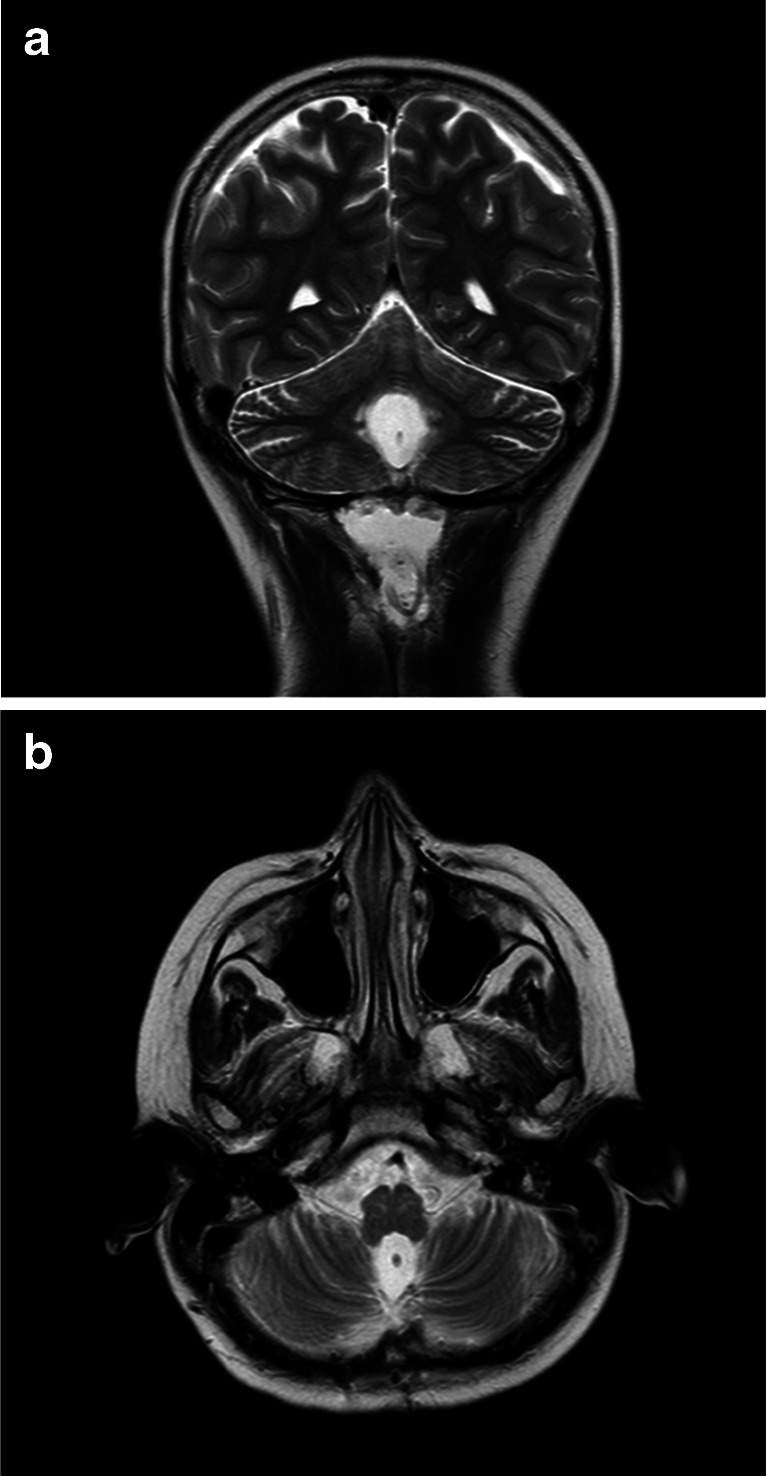
Postoperative T2-weighted MRI scans demonstrating the ventricular catheter in position in the fourth ventricle. The catheter is marked by an *arrow* in coronal (**a**) and axial (**b**) views

Postoperatively, an MRI of the brain should be obtained both to assess baseline tumor burden and to confirm the appropriate position of the catheter within the fourth ventricle. In general, the catheter is best visualized on T2-weighted MRI sequences obtained in at least two planes (Fig. 6). Additionally, prior to using the VAD to administer chemotherapy, CSF flow from the fourth ventricular outlets to the cervical, thoracic, and lumbar spine should be confirmed radiographically. At our center, this confirmation is obtained by performing CINE MRI sequences of the brain and total spine. If CSF flow from the fourth ventricle to the lumbar spine is not confirmed, then a nuclear medicine CSF flow study should be obtained.

### Performing infusions using the fourth ventricle VAD

Infusions are performed using a 25-gauge butterfly needle after sterile prep. After accessing the device, CSF is withdrawn and sent for studies as indicated. Aspiration of fluid must be performed slowly (no greater than 1 mm per min) to avoid collapsing the fourth ventricle or aspirating brain tissue into the catheter holes. Infusions of chemotherapy or other agents and subsequent flush using preservative-free normal saline are also performed slowly, at a rate no greater than 1 mm per minute, to avoid any pressure on the adjacent brainstem or cerebellum.

## Results

Seven patients underwent surgery for implantation of a VAD in the fourth ventricle. Five patients had recurrent medulloblastoma, and two patients had recurrent anaplastic ependymoma. All seven patients had progressive disease despite prior surgery, radiation therapy, and chemotherapy. The median patient age was 12 years, with a range of 4 to 19 years old.

All patients underwent successful surgical implantation of the fourth ventricular catheter and VAD. Simultaneously, three patients underwent subtotal resection of tumor located in the fourth ventricle or cerebellum, and one patient underwent simultaneous tumor biopsy. The remaining three patients did not have tumor amenable to simultaneous resection. Six patients had no new neurological deficits after surgery, and one patient had new partial left-sided facial weakness postoperatively. This patient had undergone simultaneous subtotal resection of tumor (anaplastic ependymoma) from the fourth ventricle. The postoperative seventh nerve palsy was attributed to resection of tumor adherent to the floor of the fourth ventricle, and it did not resolve for the duration of follow-up. Postoperative MRI scans in all seven patients demonstrated catheter placement within the fourth ventricle without injury to the adjacent brainstem or cerebellum. In all seven patients, CINE MRI sequences confirmed flow of CSF from the fourth ventricular outlets to the cervical, thoracic, and lumbar spinal subarachnoid spaces. Thus, nuclear medicine CSF flow studies were not performed in any patients.

Two patients never received chemotherapy infusions into the implanted VAD because their disease progressed rapidly in the interval between VAD placement and the planned first infusions. The remaining five patients underwent, collectively, a total of 240 infusions of methotrexate into the fourth ventricle as described in the published manuscript describing the pilot trial [1]. Disease responses are detailed in this publication. None of the five patients who underwent infusions had any new neurological deficits related to these infusions, and none had leukoencephalopathy or other damage to the brainstem, cerebellum, or cerebral cortex on follow-up MRI scans after infusions.

## Discussion

VAD placement into the frontal horn of the lateral ventricle is a common procedure familiar to neurosurgeons. VAD placement into the fourth ventricle for the purpose of chemotherapy infusions, however, has not been reported prior to the recently published clinical trial from our center [1]. This publication focused on details of the clinical trial and its results and did not include the technical aspects of VAD placement. The purpose of the current manuscript is to describe in detail, for an audience of neurosurgeons, the technical nuances required to safely place and utilize a fourth ventricle VAD.

Catheter and VAD placement into the fourth ventricle in children with posterior fossa tumors may have advantages over the more traditional means of administering intrathecal chemotherapy via lumbar puncture or intraventricular chemotherapy via lateral ventricle VAD. Lumbar punctures often require sedation in young children, are technically difficult in some patients, and are more painful than VAD access. In prior studies, intrathecal infusions via lumbar puncture yielded lower and less consistent ventricular drug levels than intraventricular infusions [2, 3]. Lateral ventricle VAD placement is most commonly a separate surgical procedure performed on a different date than initial posterior fossa craniotomy for tumor resection, typically at the time of recurrence. By placing a fourth ventricle VAD at the time of tumor resection, a separate surgical procedure and anesthesia administration can be avoided. Additionally, fourth ventricle catheters are placed under direct vision using the operating microscope rather than blindly through cerebral cortex. Catheter placement without passing through any brain parenchyma may potentially decrease the risk of intracerebral hemorrhage and catheter malposition that can occur with lateral ventricle catheter placement [4–7]. While frameless stereotaxy can minimize catheter malposition with lateral ventricle VAD placement, it cannot guarantee that all catheter holes are within the ventricle rather than cerebral cortex, especially in patients with small lateral ventricles. When some catheter holes are within cerebral cortex, infusion of chemotherapeutic agents directly into this cortex may contribute to leukoencephalopathy [4, 8–11]. While the sample size of our clinical trial is small, it is notable that none of the patients who received infusions had any radiographic or clinical evidence of leukoencephalopathy after chemotherapy infusions [1]. A larger patient sample will be required to determine if fourth ventricle VAD placement can consistently avoid this complication.

While an additional surgery and some complications may possibly be avoided by placing a fourth ventricle VAD, the technical nuances described here are important tips to avoid complications. If the catheter is not placed and firmly secured in such a way that it does not touch the brainstem or cerebellum, injury to these structures is possible. The catheter must be secured such that it does not migrate, but neck movement is allowed without tension on the catheter. When utilizing the catheter, it is important to aspirate CSF slowly to avoid creating contact between the catheter and adjacent brainstem or cerebellum. Similarly, infused agents should be administered slowly to avoid causing any pressure on the brainstem.

Fourth ventricle VADs may not be the ideal means of administering intraventricular chemotherapy to all patients with malignant tumors originating in the posterior fossa. When metastatic lesions are present in the lateral and/or third ventricles, higher drug levels may potentially be achieved in these compartments with a lateral ventricle catheter. The recently reported pilot trial from our center enrolled only patients with recurrent disease who had failed standard therapies [1]. However, placement of a VAD into the fourth ventricle for administration of chemotherapy or novel agents may be most logical at the time of initial resection when the only remaining known disease is tumor that is adherent to the floor of the fourth ventricle. Previous animal studies suggest that drug administration into the fourth ventricle at that time would yield high drug levels at the site of residual disease as well as regional distribution to CSF spaces throughout the neuraxis [12–14]. Future studies will include placement of VADs into the fourth ventricle at the time of initial surgical resection for administration of intraventricular chemotherapy postoperatively in selected patients.

## Conclusions

Fourth ventricle VADs are not currently utilized at the majority of centers that treat children with brain tumors. However, ongoing clinical trials may lead to increased interest in direct delivery of chemotherapy and novel agents into the fourth ventricle after resection of brain tumors that originate in this location. Technical nuances described in this manuscript will facilitate safe placement and utilization of fourth ventricle VADs.
